# Unravelling the reactive oxygen and reactive nitrogen signalling networks in plants

**DOI:** 10.1093/jxb/erv124

**Published:** 2015-05-07

**Authors:** Luisa María Sandalio, Christine Helen Foyer

**Affiliations:** CSIC, Estación Experimental del Zaidín, Spain; University of Leeds, UK

Plants are the basis of life on earth, generating oxygen from water to replenish the atmospheric oxygen required for aerobic respiration, as well as fixing atmospheric carbon to provide food and fuel that animals and humans need to survive. Photosynthesis is recognized as a key process in the evolution of aerobic life forms that not only allows carbon, oxygen, nitrogen, and other nutrients to move through ecosystems in a predictable manner but also serves as a paradigm for reduction–oxidation (redox) control and the regulated generation of reactive oxygen species (ROS). This type of control is particularly important in plant responses to stressful environmental conditions.

Plants are continuously exposed to changing environmental conditions, many of which such as drought, salinity, high and low temperatures, heavy metals, and insect and pathogen attack cause ‘stress’ to the plant, limiting vigour and crop yields, with negative impacts on agronomy, human health and well-being. It is perhaps not surprising, therefore, that they have evolved a complex network of physical, metabolic, and genetic mechanisms that enable them to cope with external threats and challenges, including those imposed by the actions of humans, such as climate change and pollution.

Most, if not all, biotic and abiotic stress conditions lead to changes in cellular redox homeostasis with increased ROS production and accumulation, and altered nitric oxide (NO) production and metabolism. ROS are produced by many other metabolic processes in addition to photosynthesis, particularly enzymes that are activated in times of stress to produce superoxide and hydrogen peroxide in a characteristic ‘oxidative burst’. ROS accumulation in such circumstances is often considered to underpin the phenomenon called oxidative stress. Like the gaseous signalling molecule NO, ROS are common intracellular and intercellular messengers with a broad spectrum of regulatory functions in many physiological processes. In his review, **Luis A del Río**, a pioneer of ROS chemistry in plants has provided a comprehensive description of the history of research and advances that underpin our current knowledge and understanding of ROS and NO in plants, highlighting the major contributions made by different researchers and scientific groups. ROS and NO play important roles in the toxicity caused by heavy metals and in the metabolic disturbances caused by perturbations in nutritional status. The low-molecular-weight thiol antioxidant, reduced glutathione (GSH), has multiple functions in plants and it is particularly important in plant defences against xenobiotic and heavy metals, as discussed in detail by **Hernandez *et al*.** In addition, new evidence supports a role for the respiratory ALTERNATIVE OXIDASE1a in modulating oxidative stress in plants exposed to cadmium (**Keunen *et al*.**) and this paper provides new evidence for the crucial role of mitochondria in plant responses to metals. Like mitochondria, chloroplast processes are important in many plant stress responses. The timely review by **Hernández and Munné-Bosch** provides novel insights into the role of photo-oxidative stress in the chloroplasts that is associated with phosphorous deficiency.

NO and its derivatives, particularly reactive nitrogen species (RNS), interact with ROS and participate in plant responses to biotic and abiotic stresses. Although gene encoding classic nitric oxide synthase (NOS) enzymes exist in plant genomes, NO is produced by both enzymatic and non-enzymatic sources in plants. NO and RNS act together with ROS to regulate a wide range of physiological and developmental processes such as stomatal closure, germination, root development, gravitropism, and programmed cell death (PCD). An interesting example of ROS/NO/RNS that prevents self-fertilization and promotes genetic variability is described in the review by **Serrano *et al*.** A timely and up-to-the-minute description of our current knowledge concerning the pathways of ROS signalling, particularly through MAP kinase cascades, and the interplay between ROS and plant hormone signalling pathways is provided in the review by **Xia *et al*.** Similarly, a comprehensive account of the extensive network of ROS, NO, and phytohormone interactions in the control of plant development and stress tolerance is provided by **Sanz *et al*.**

Considerable progress has also been made in our knowledge of the ROS/NO communication that underpins symbiotic interactions between leguminous plants and nitrogen-fixing *Rhizobia*. The paper by **Hichri *et al.*** provides new information on NO signalling during the establishment of this symbiosis, together with the multi-faceted functions of NO in the regulation of symbiotic nitrogen-fixation. In addition, the paper by **Matamoros *et al.*** provides exciting new information on the roles of glutathione peroxidases in nodule establishment and function. GSH plays important functions in the nucleus and cytosol during the cell division that underpins the formation of new organs such as nodules. The role of the nuclear GSH pool is described in relation to the action of the cell cycle inhibitor, ophiobolin A, in the paper by **Locato *et al*.** In this study, the addition of ophiobolin A, which is a sesterpenoid toxin produced by pathogenic fungi, is shown to cause arrest of the cell cycle at the G2/S phase and to alter the profile of glutathionylated and ADP-ribosylated proteins even though glutathione is retained in the nucleus.

The regulated changes in ROS and NO/NRS production and accumulation are associated with wide range of post-translational modifications to proteins including carbonylation, glutathionylation, cysteine oxidation to sulphenic, sulphinic, and sulphonic acids, nitration, and *S*-nitrosylation, which act as metabolic switches regulating plant responses to environmental changes. These post-translational modifications function as metabolic switches in the cell signalling pathways that underpin plant stress responses. However, excessive cellular oxidation and/or NO accumulation lead to high levels of irreversible protein oxidation, nitration or *S*-nitrosylation. **Correa-Aragunde *et al*.** have provided a comprehensive description of NO-dependent post-transcriptional modifications and their roles in cellular redox homeostasis, with particular focus on the regulation of *S*-nitrosylation of the enzyme ascorbate oxidase. The importance of cysteine residues in such post-translational modifications is reviewed by **Akter *et al*.** and **Waszczak *et al*.** They discuss the different oxidized forms of cysteine found in proteins, together with the proteomic approaches that are currently being used to study these modifications.

The accumulation of ROS and/or NO in a particular cellular location or at a given time is regulated by a subtle balance between the rates of production and scavenging/processing by antioxidant defences/NO metabolism. The tempo-spatial accumulation of NO and ROS are considered to be important in conferring specificity to the signals underpinning plant responses to a given stimulus. However, the mechanisms by which cellular stress-perception systems regulate the signalling microenvironments and form discrete niches for specific ROS and NO production remain largely unresolved. The review by **Sevilla *et al*.** provides a concise overview of our current knowledge of the thioredoxin/peroxiredoxin/sulphiredoxin systems in different cellular compartments and their respective roles in regulating different protein targets, processes that might also underpin cell signalling. Moreover, the paper by **Puerto-Galán *et al*.** provides new information on the contribution of NADPH thioredoxin reductase C and the sulphiredoxin to 2-Cys peroxiredoxin overoxidation to chloroplast regulation.

The last five years have seen progress that has increased our understanding of the extensive cross-talk between ROS, NO, cellular redox changes, and hormone-mediated pathways. However, many questions remain. For example, even though the level of NO appears to be efficiently regulated in plant cells, little information is available concerning how the different mechanisms of NO production are integrated or how NO is scavenged or processed. Our incomplete knowledge of these systems and their regulation constitute significant bottlenecks to progress. One of the major challenges over the next ten years is to decipher the complexity of ROS and NO functions of biotic and abiotic stress signalling pathways particularly in terms of spatio-temporal regulation.

The explosion of research interest and publications observed over the last 25 years in the multifaceted area of redox biology shows no signs of slowing down. Moreover, new avenues of research in this field continue to open, particularly, for example, in topics such as the redox-dependent genetic and epigenetic controls of target genes and the interplay between different post-translational mechanisms of protein regulation.

